# Vitamin k3 inhibits protein aggregation: Implication in the treatment of amyloid diseases

**DOI:** 10.1038/srep26759

**Published:** 2016-05-27

**Authors:** Parvez Alam, Sumit Kumar Chaturvedi, Mohammad Khursheed Siddiqi, Ravi Kant Rajpoot, Mohd Rehan Ajmal, Masihuz Zaman, Rizwan Hasan Khan

**Affiliations:** 1 Molecular Biophysics and Biophysical Chemistry Group, Interdisciplinary Biotechnology Unit, Aligarh Muslim University, Aligarh-202002, India; 2 Recombinant Gene Product Group, International Centre for Genetic Engineering and Biotechnology, Aruna Asaf Ali Marg, New Delhi-110067, India

## Abstract

Protein misfolding and aggregation have been associated with several human diseases such as Alzheimer’s, Parkinson’s and familial amyloid polyneuropathy etc. In this study, anti-fibrillation activity of vitamin k3 and its effect on the kinetics of amyloid formation of hen egg white lysozyme (HEWL) and Aβ-42 peptide were investigated. Here, in combination with Thioflavin T (ThT) fluorescence assay, circular dichroism (CD), transmission electron microscopy and cell cytotoxicity assay, we demonstrated that vitamin k3 significantly inhibits fibril formation as well as the inhibitory effect is dose dependent manner. Our experimental studies inferred that vitamin k3 exert its neuro protective effect against amyloid induced cytotoxicity through concerted pathway, modifying the aggregation formation towards formation of nontoxic aggregates. Molecular docking demonstrated that vitamin k3 mediated inhibition of HEWL and Aβ-42 fibrillogenesis may be initiated by interacting with proteolytic resistant and aggregation prone regions respectively. This work would provide an insight into the mechanism of protein aggregation inhibition by vitamin k3; pave the way for discovery of other small molecules that may exert similar effect against amyloid formation and its associated neurodegenerative diseases.

Amyloid fibrillation of proteins and peptides are responsible for wide number of diseases in humans such as Alzheimer’s, Parkinson’s, Huntington’s, and Diabetes II etc^1^,^2^. Despite the unrelated amino acid sequences and tertiary structure, proteins can unfold and assemble to form amyloid fibrils with similar ultrastructure and biochemical properties^3^. Amyloid fibrils do have long unbranched shape, enriched beta sheet content, cytotoxic nature, high surface hydrophobicity and specific tinctorial properties^4^. Although, the propensity to form amyloid fibrils is modulated by polypeptide sequence and varies from sequence to sequence^5^.

HEWL is a small size, 129 amino acid residues long protein that has been used as a model to study the mechanism of amyloid formation and inhibition^6^. Further, HEWL is homologous to human lysozyme which is responsible for non-neuropathic systemic amyloidosis resulting in deposition of amyloids in liver and kidney in humans. Alzheimer’s diseases is one of the most common form of dementia, fibrillar aggregates of amyloid beta peptides of 39–43 amino acids is the main factor responsible for the disease. The amyloid cascade hypothesis presumes that amyloid aggregates self-assembled from misfolded Aβ peptides, affect the structure and function of neuronal cells as well as stimulate apoptosis that is responsible for synaptic dysfunction and neurodegeneration^7^.

Currently, many methodologies have been developed to find suitable cure for amyloid associated diseases and is still progressing. Small molecules/drugs which can stabilize the native state of protein retard the fibrillation process or reverse the misfolding process can serve as promising agent against debilitating diseases^8^. Many compounds such as polyphenols, various drugs, flavonoids, vitamins and some metal complexes are known to inhibit the aggregation process of proteins either *in vitro* or *in vivo*^9^,^10^,^11^,^12^. Recently numbers of reports are available advocating the anti-aggregation effect of nanomaterial’s^13^,^14^,^15^,^16^. Varieties of vitamins including vitamin A, vitamin E, vitamin D and vitamin k attenuated the aggregation process in proteins and also delayed the onset of dementia^17^,^18^,^19^,^20^,^21^. In order to get further insight into the mechanism of vitamins mediated aggregation inhibition of proteins; we evaluated the effect of vitamin k3, a synthetic analogue of vitamin k on fibrillation of HEWL and Aβ-42 peptide.

Vitamin k3, an anti-inflammatory and anti-cancer agent, is employed for post translational modification of proteins including blood clotting factors. Similar behaviour to vitamin k makes it a good choice as a health supplement and component of multivitamin drugs^22^,^23^,^24^.

In this study, mechanism of action of vitamin k3 has been investigated on HEWL and Aβ-42 amyloid fibrillogenesis process by exploiting various complementary techniques. To assess the effect of vitamin k3 on the growth phase of amyloid, aggregation kinetics study was performed by ThT binding assay. Changes in secondary structure were examined by CD spectroscopy. Hydrodynamic radii were monitored by using dynamic light scattering measurements. Morphology of aggregates was studied by using transmission electron microscopy. Interaction mode of vitamin k3 with HEWL and Aβ-42 was studied using molecular docking. Further, cell viability (MTT) assay was accomplished by using SH-SY5Y cell lines.

## Results and Discussion

### ThT Binding Assay

**Figure 1:**
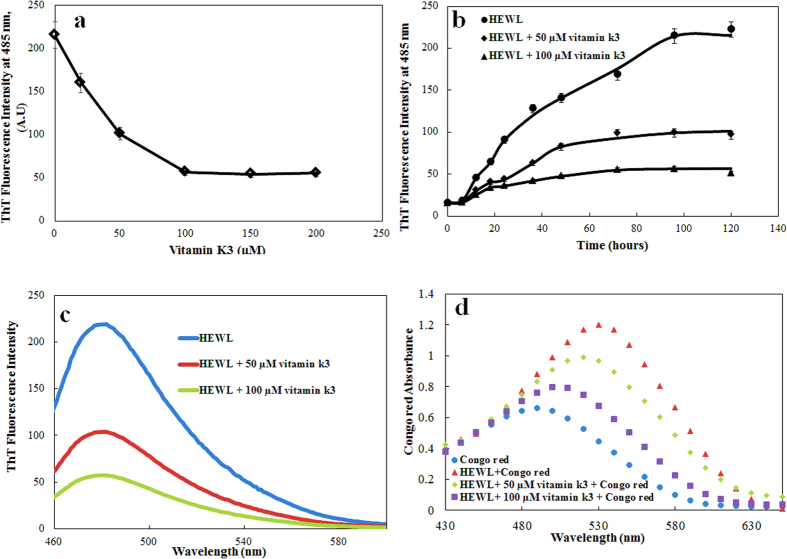
(**a**) ThT fluorescence intensity of HEWL (15 μM) at 485 nm in presence of varying concentration of vitamin k3 (0–200 μM). Samples were incubated at 65 °C for 120 hours. (**b**) ThT fluorescence kinetics of HEWL in absence and presence of vitamin k3 (50 and 100 μM) (**c**) ThT fluorescence spectra of HEWL incubated at 65 °C over 120 hours in absence and presence of vitamin k3 (50 and 100 μM) (**d**) Congo red binding absorption spectra of HEWL (15 μM) in the absence and presence of vitamin k3. Samples were incubated with two different concentration of vitamin k3 (50 and 100 μM) at 65 °C for 120 hours. Experimental data represent the average ± s.d (n = 3).

Thioflavin T (ThT) fluorescence assay was carried out to quantify amyloid formation of HEWL aggregates and examining the impact of vitamin k3 on rate of fibril formation. ThT fluorescence has been widely used to detect cross β- sheet structure of amyloids^25^. The growth of amyloid formation of HEWL was monitored and characterized by ThT. On interaction with cross- β sheet structure, ThT results in significant rise in fluorescence intensity^26^,^27^. The inhibitory effect of vitamin k3 on fibrillation process was concentration dependent (Fig. 1a). Kinetics of amyloid formation in the absence and presence of vitamin k3 (50 μM and 100 μM) is shown in Fig. 1b. As shown in Fig. 1b, the aggregation kinetics of HEWL exhibit a typical sigmoidal appearance containing a lag phase associated with nucleation, a fast growth phase linked to the elongation and propagation of fibrils, and a final stationary phase^28^. Further, these results are in agreement with the nucleation dependent polymerisation for amyloidogenic proteins. However, simultaneous incubation of HEWL with 50 and 100 μM vitamins k3 significantly attenuated the ThT fluorescence intensity throughout the time frame of the experiment (Fig. 1b). ThT fluorescence spectra of HEWL and HEWL in combination with vitamin k3 after 120 hours incubation at 65 °C are shown in Fig. 1c. It can be seen from the figure that ThT fluorescence intensity of HEWL was reduced from 222 to 96 and 50 in presence of 50 and 100 μM of vitamin k3 respectively. Further addition of vitamin k3 to preformed HEWL amyloid fibrils did not lead to decrease in ThT fluorescence intensity, it suggests that vitamin k3 cannot reverse the fibril formation but can only inhibit its formation. These results revealed that vitamin k3 inhibits fibrillation from very beginning of log phase as observed in the case of EGCG which inhibits fibrillogenesis of Chicken Cystatin^29^.

### Congo red binding assay

To corroborate the findings of ThT assay, the inhibition of HEWL aggregation was also confirmed by Congo red binding assay. A red shift in absorbance maxima of Congo red absorption spectrum from 490 nm to 540 nm indicates presence of increased cross β-sheet rich structure^30^. HEWL samples incubated for 120 hours at 65 °C showed marked increase in absorbance accompanied with red shift in comparison to Congo red alone, signifying the presence of amyloid fibrils as represented in Fig. 1d. We observed that this increased peak shift in HEWL was prevented in presence of vitamin k3 and more pronounced effect was observed in presence of 100 μM^31^. These results also in accord with preceding ThT fluorescence results suggesting that fibril formation is inversely related to the concentration of vitamin k3.

### Surface hydrophobicity modulation by vitamin k3

**Figure 2:**
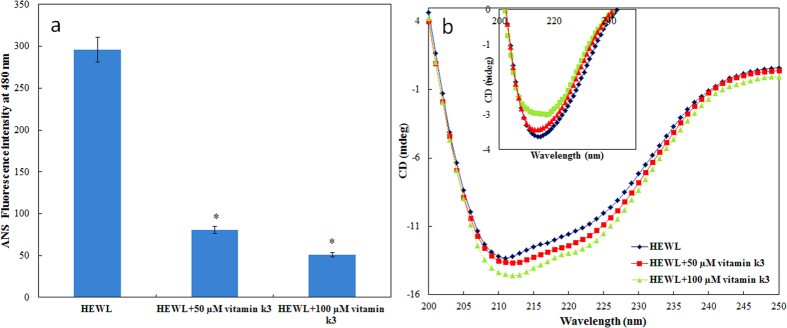
(**a**) ANS fluorescence intensity of HEWL (15 μM) at 480 nm incubated at 65 °C over 120 hours in absence and presence of vitamin k3 (50 and 100 μM). *Statistically different from the HEWL p ≤ 0.01. (**b**) Far-UV CD spectra of HEWL (15 μM) at 25 °C in absence and presence of vitamin k3. Inset (**b**) shows HEWL (15 μM) incubated at 65 °C over a period of 120 hours in the absence and presence of vitamin k3 (50 and 100 μM).

The impacts of vitamin k3 on surface hydrophobicity of HEWL were examined based on ANS fluorescence emission. ANS is widely used to characterise the protein folding intermediate states and to detect the presence of hydrophobic patches on the surface of protein/amyloids^32^. Interaction of ANS with solvent exposed hydrophobic patches on the protein leads to significant increase in fluorescence intensity^33^. The surface hydrophobicity of HEWL at 25 °C with or without vitamin k3 was found to be very low because in native protein hydrophobic patches are hidden inside in compactly folded structure (data not shown). In contrast, a marked increase in ANS fluorescence intensity was observed in HEWL upon incubation at 65 °C for 120 hours (Fig. 2a)^34^. Conversely, low ANS fluorescence intensity was observed in HEWL-vitamin k3 (50 and 100 μM) co-incubated samples. This suggests that exposure of hydrophobic regions is significantly decreased in presence of vitamin k3. The reduced ANS fluorescence intensity observed in presence of vitamin k3 which indicates less exposer of hydrophobic core to solvent, suggested that vitamin k3 could stabilise the whole conformation of HEWL even though it was subjected to at high temperature. It is believed that non covalent bonds stabilize the core structure of all amyloid fibrils^35^. Vitamin k3 might have interacted via non covalent interaction with amino acid residues and interfere with the formation of HEWL fibrils.

### Conformational transition in HEWL during fibrillation

CD is well known spectroscopic technique to elucidate the secondary structure of proteins^36^. The conversion of alpha helix or random coil to beta sheet is hallmark of amyloid formation^37^. The CD spectra of HEWL obtained prior to incubation exhibited the typical profile of predominantly alpha helical conformation and in presence of vitamin k3, increased in alpha helical content from 32 to 41% was observed at 25 °C (Fig. 2b)^38^. As was expected, the fibrillation of HEWL while incubating at 65 °C for 120 hours resulted in decreased of alpha helical fraction and concomitant increase in beta sheet fraction (inset Fig. 2b and supplementary Table 1). Although we observed single minima at around 218 nm in HEWL- vitamin k3 co-incubated samples but the negative CD value at this point was reduced. To calculate the secondary structure composition of each CD spectra, k2d3 software was used for calculating various secondary structure components (alpha helix, beta sheet and random coil)^39^. The anticipated decrease in beta sheet content was observed in presence of vitamin k3 in HEWL. These results suggest that structural transition in HEWL was mitigated upon co-incubation with vitamin k3^40^.

### Dynamic light scattering measurements

**Figure 3:**
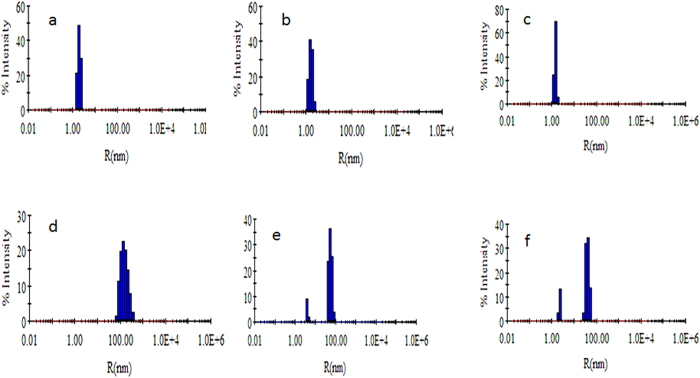
DLS pattern of HEWL in absence and presence of vitamin k3 at 25 °C (**a**) HEWL (**b**) HEWL + 50 μM vitamin k3 (**c**) HEWL + 100 μM vitamin k3 and after incubation at 65 °C over a period of 120 hours (**d**) HEWL (**e**) HEWL + 50 μM vitamin k3 and (**f**) HEWL + 100 μM vitamin k3.

In order to determine the size of protein aggregates formed in the absence and presence of vitamin k3, dynamic light scattering measurements were performed. Results are summarised in Fig. 3. It is quite apparent from figure that hydrodynamic radii (*R*_*h*_) of HEWL at 25 °C decreases slightly (2 nm to 1.8 nm) in presence of vitamin k3. Decrease in *R*_*h*_ might be attributed to disturbance of solvation sphere around protein suggesting that vitamin k3 stabilize the native state of protein which is in consistent with our CD data. The *R*_*h*_ value of HEWL increases with respect to time upon incubation at 65 °C but in presence of vitamin k3, concentration dependent decrement in *R*_*h*_ was observed. HEWL incubated for 120 hours shows *R*_*h*_ value around 400 nm but in presence of vitamin k3 this value decrease to 40–60 nm. This decrease in *R*_*h*_ value might be attributed to the stabilization of protein in presence of vitamin k3.

### Transmission electron microscopy

**Figure 4:**
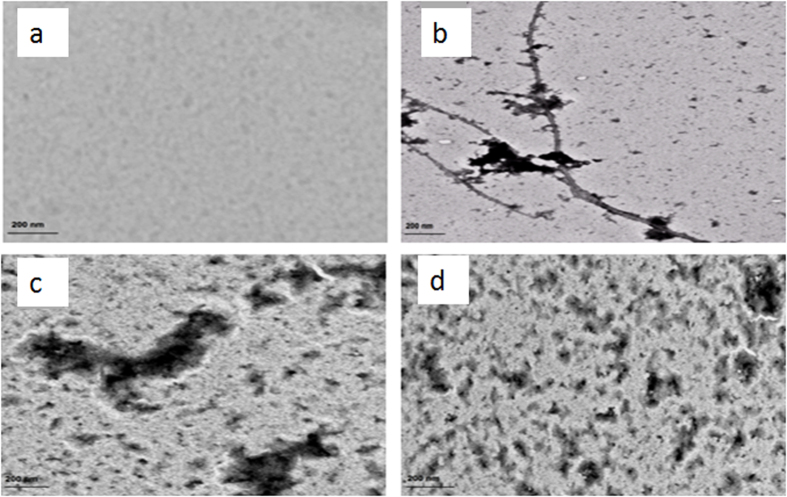
Transmission Electron Microscopic images (**a**) HEWL at 25 °C (**b**) HEWL incubated at 65 °C over 120 hours (**c**) HEWL + 50 μM vitamin k3 incubated at 65 °C over 120 hours (**d**) HEWL + 100 μM vitamin k3 incubated at 65 °C over 120 hours.

To further investigate the efficacy of vitamin k3 against HEWL fibril formation, TEM analysis was performed. As shown in Fig. 4, HEWL sample alone incubated for 120 hours formed large, branched fibrils which are characteristic feature of amyloids^41^. Samples of HEWL in presence of vitamin k3 (50 and 100 μM) showed no fibrillar aggregate. This result reveals that vitamin k3 inhibits aggregation process of HEWL in concentration dependent manner. The possible mechanism for vitamin k3 mediated reduction in HEWL aggregation may be coercing of the protein in its native state^34^. This observation is similar to previous studies on the effect of osmolytes on insulin fibrillation^42^.

### Vitamin k3 reduces amyloid inflicted cytotoxicity

**Figure 5:**
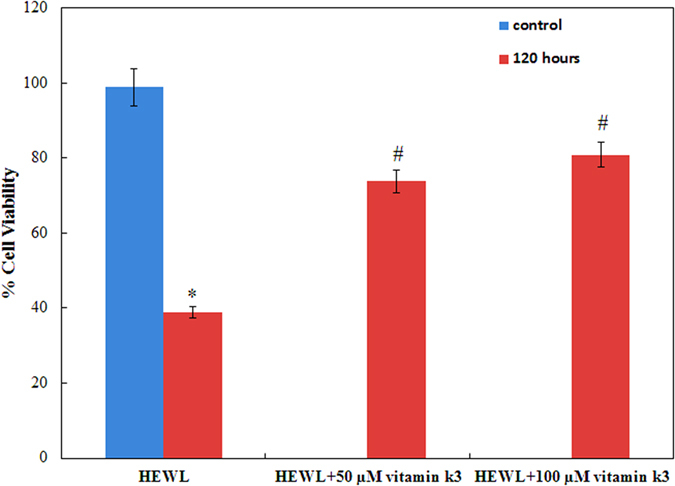
MTT reduction assay for cell cytotoxicity of 120-hours aged HEWL amyloid fibrils in absence and presence of vitamin k3 (50 and 100 μM) on SH-SY5Y cell lines. Control represents the cells without exposed to HEWL fibrils. *Statistically different from the control group, p ≤ 0.01 and ^#^statistically different from the HEWL, p ≤ 0.01.

To investigate whether HEWL amyloids are cytotoxic or not and if this cytotoxicity could be relieved by adding vitamin k3, we employed SH-SY5Y (human neuroblastoma) cell line for cell viability assay at 24 hours. When vitamin k3 (50 and 100 μM) added to SH-SY5Y cell line showed no cytotoxic effect (data not shown). Further vitamin k3 added to preformed amyloids have no protective effect on cytotoxicity, ruling out other protective mechanism apart from aggregation inhibition (data not shown). SH-SY5Y cell lines were treated with 120 hours aged HEWL amyloids and their effect on cell viability was checked and data is represented in Fig. 5. It is inferred from the figure that cell viability decreases in presence of 120 hours aged HEWL amyloid and reaches up to 38%^34^. Further, it is clear from the cytotoxicity profile that HEWL incubated with vitamin k3 enhance cell viability, even in presence of 120 hour aged amyloid. Cell viability was rescued to 73% and 80% in presence of 50 μM and 100 μM vitamin k3 respectively. Cell toxicity of HEWL may be attributed to the formation of amyloids which disrupts the cell membrane. In presence of vitamin k3, gain in cell viability was observed. This confirmed that increase in cell viability was due to anti amyloidogenic behaviour of vitamin k3^43^. These results suggest that the non fibrillar aggregates formed in the presence of vitamin k3 are less toxic to neuronal cells therefore related compounds may have therapeutics intervention against amyloids diseases^44^.

### Binding mode of vitamin k3 to HEWL

Based on the spectroscopic and electron microscopy data it can be inferred that vitamin k3 inhibits amyloid formation of HEWL. Information on putative binding site and mode of interaction between HEWL and vitamin k3 was obtained from molecular docking using Autodock 4.2 program^45^. Molecular docking results are summarises in supplementary Figure S1 and supplementary Table 2. It is clear from the figure that vitamin k3 interact with HEWL with the following amino acid residues Lys^1^, Phe^3^, Glu^7^, Ala^10^, Ala^11^, Arg^14^, His^15^, Thr^40^, Gln^41^, Leu^84^, Ser^85^, Ser^86^, Asp^87^ and IIe^88^. The main interaction forces between HEWL and vitamin k3 are hydrophobic interaction. It is clear from these results that vitamin k3 interact with proteolitically resistant region (32–108). Interaction with this region might be responsible for anti amyloidogenic behavior. Inspite of no interaction with amyloid prone region of HEWL vitamin k3 inhibits aggregation similar to inhibitory action of nanobody on aggregation of human lysozyme^34^. As generally acknowledged that protein aggregate mostly through hydrogen bonding, aromatic and hydrophobic interaction, disrupting of such interactions by vitamin k3 may thus inhibits amyloid formation^46^. The value of Gibbs free energy for the best pose was −3.98 kcal mol^−1^, that suggests the formation of a stable protein–ligand complex^47^. Our results implied that the inhibition of HEWL fibrillogenesis can be attributed mainly to the binding of vitamin k3 to HEWL proteolitically resistant region.

### Vitamin k3 inhibits amyloid fibrillation of Aβ peptide

**Figure 6:**
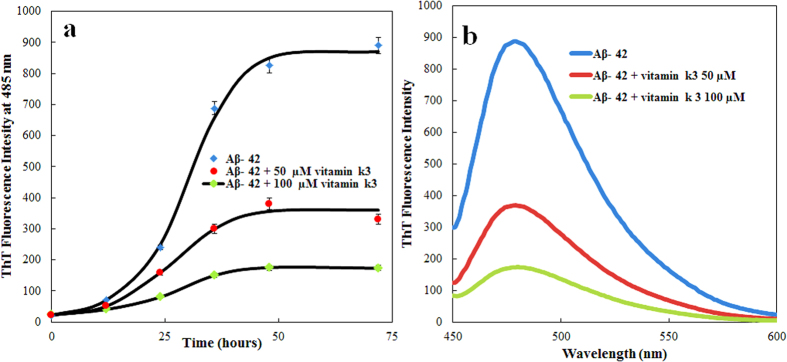
(**a**) ThT fluorescence kinetics measurement of Aβ-42 aggregation when incubated at 37 °C over a period of 70 hours in absence and in presence of vitamin k3 (50 and 100 μM) (**b**) ThT fluorescence spectra of Aβ-42 after incubation at 37 °C for 70 hours in absence and in presence of vitamin k3 (50 and 100 μM). Experimental data represent the average ± s.d (n = 3).

Inhibition of Aβ-42 fibrillation was also examined in presence of vitamin k3 (50 and 100 μM). Figure 6a,b represented the ThT kinetics of Aβ-42 aggregation and ThT fluorescence intensity of 70 hours aged amyloids respectively, in the absence and presence of vitamin k3. Aβ-42 aggregates gave strong ThT emission, producing a typical sigmoidal curve that reaches the plateau stage after almost 50 hours incubation. As predicted this observed behavior was opposite to that we observed when Aβ-42 incubated in presence of vitamin k3. Under these conditions the kinetic of Aβ-42 aggregation was delayed and initiated only after 25 hours and 40 hours of incubation in presence of 50 and 100 μM of vitamin k3 respectively. Addition of vitamin k3 significantly inhibited the aggregation of Aβ-42 which is in well agreement with previous reports delineating the effect of small molecules on aggregation^48^.

Congo red binding assay was used as an indicator of extended β-pleated sheet structures and Aβ-42 aggregation. Congo red absorption spectra of Aβ-42 in absence and presence of vitamin k3 is shown in supplementary Figure S2a. Protein solution containing amyloid fibrils exhibited an increase in Congo red absorption accompanied with a red shift of the spectral maximum. Analogous to ThT fluorescence results, co-incubation of Aβ-42 with vitamin k3 resulted in both reduction of absorbance intensity and also blue shift in wavelength maximum.

ANS has long been used as fluorescent molecular probe for examining hydrophobicity of proteins. ANS binding was used to study the changes in hydrophobicity in Aβ-42 upon incubation in absence and presence of vitamin k3. The ANS fluorescence maxima in absence and presence of vitamin k3 are shown in supplementary Figure S2b. It is clear from the figure that Aβ-42 alone shows very high ANS binding suggesting exposure of hydrophobic patches in contrast the ANS binding was significantly reduced in presence of vitamin k3. Similar observation was made by Ghaghaei *et al*. in the protective role of crocin in Aβ-42 fibril formation^49^.

In order to detect the change in the secondary structure, CD spectroscopy in the far UV region (200–250) was carried out for Aβ-42 in absence and presence of vitamin k3. CD spectra of native Aβ-42 displayed characteristic spectra of random coil structure of the peptide (supplementary Figure S3) but almost no structural change was observed in the peptide at 0 hour in the presence of vitamin k3 (data not shown). As shown in figure 70 hour of incubation at 37 °C a negative peak at around 218 nm was observed suggesting the structural transition from random coil to beta sheet in the peptide. But the structural conversion from random coil to beta sheet was retarded by vitamin k3 manifested by decrease negative band at around 218 nm as compared to native Aβ-42. These results indicate that vitamin k3 inhibits Aβ-42 aggregation by decreasing the beta sheet formation. Similar results were observed by Dai *et al*. for Chitosan Oligosaccharides inhibition of Aβ-42 aggregation^50^.

Dynamic light scattering measurements was employed to get qualitative estimation of the size of Aβ-42 aggregates. Aβ-42 peptide without incubation has hydrodynamic radii (*R*_*h*_) of 4 nm as shown in supplementary Figure S4 and almost no change in *R*_*h*_was observed in presence of vitamin k3 (date not shown)^51^. But after incubation of Aβ-42 for 70 hour at 37 °C results in increase in hydrodynamic radii to around 550 nm. In contrast when Aβ-42 was incubated with vitamin k3 the *R*_*h*_ value was found to be between to 35–55 nm (Fig. S4). This result further indicates that vitamin k3 inhibits Aβ-42 aggregation.

**Figure 7:**
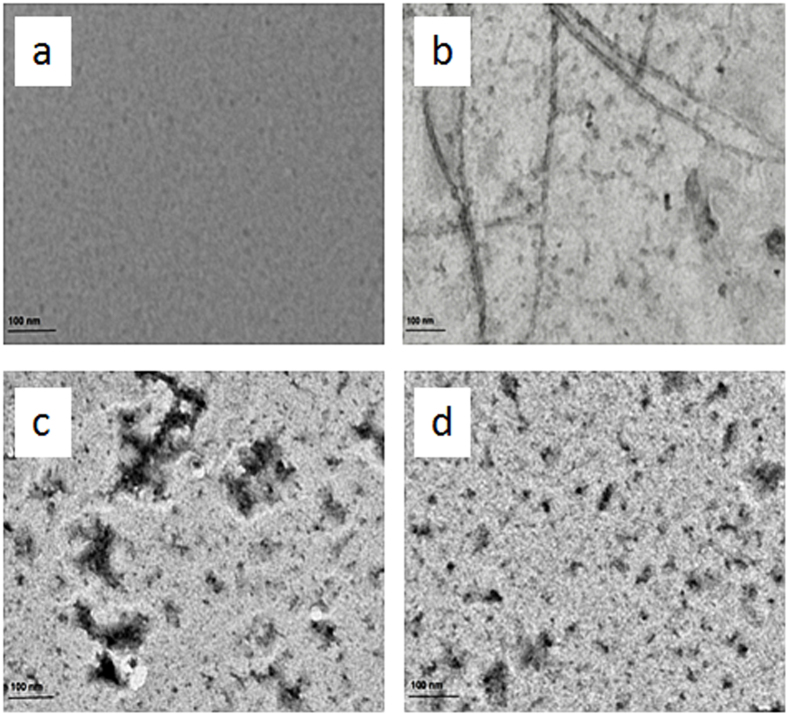
Transmission Electron Microscopic images of (**a**) Aβ-42 at 25 °C (**b**) Aβ-42 incubated for 70 hours at 37 °C (**c**) Aβ-42 + 50 μM vitamin k3 incubated for 70 hours at 37 °C (**d**) Aβ-42 + 100 μM vitamin k3 incubated for 70 hours at 37 °C.

TEM was performed to determine the morphology of aggregates formed. It is clear from the Fig. 7 that control Aβ-42 samples shows long branched fibrils which are characteristic feature of amyloids^52^. As compared to control samples in presence of vitamin k3 short and sparsely populated non fibrillar aggregate were formed. Based on TEM images, our results thus indicate that incubation of Aβ-42 with vitamin k3 inhibit Aβ-42 aggregation and significantly alter the morphology of Aβ fibrillar aggregates.

### Vitamin k3 reduce amyloid beta inflicted cell cytotoxicity

**Figure 8:**
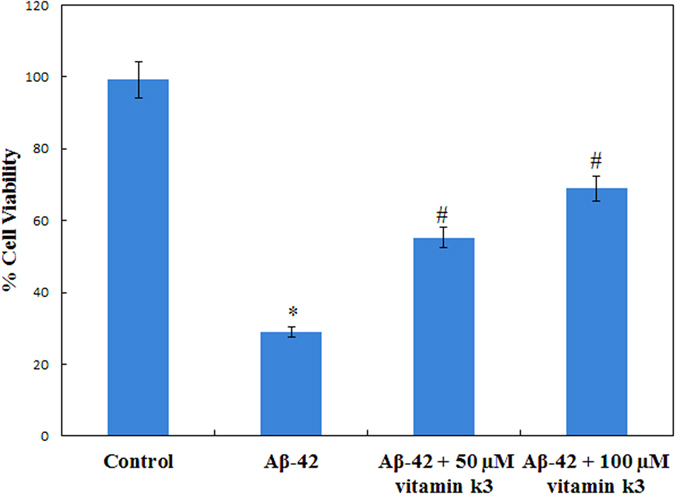
SH-SY5Y cell viability after being exposed to 70 hours aged Aβ-42 fibrils formed in the absence and presence of (50 and 100 μM) vitamin k3. *Statistically different from the control group, p ≤ 0.01 and ^#^statistically different from the Aβ-42, p ≤ 0.01.

Cultured human neuroblastoma cells (SH-SY5Y) were treated with an aliquot of Aβ-42 with and without vitamin k3 and cell viability was assessed by MTT assay, which measures metabolic activity. As shown in Fig. 8, Aβ-42 amyloids significantly reduce cell viability approximately up to 28% in SH-SY5Y cells. However Aβ-42 co-incubated with vitamin k3 (50 and 100 μM) showed higher level of viability suggesting its chemo preventive role against Aβ-42 aggregates induced cytotoxicity. It has been reported previously that exposure of neurons to β- amyloid induces degeneration and cell death involving apoptotic pathway that may contribute to the neuronal loss associated with Alzheimer disease^53^. Similar mechanism may involve in cell degeneration in the present study after exposure to Aβ-42 amyloid and our data suggest that vitamin k3 prevented this cellular degeneration. Previous works suggest that β- amyloid induced cytotoxicity could be mediated by oxidative stress and free radicals. It is known that vitamin k3 has free radical scavenging and anti-oxidant activity and this could inhibit the Aβ-42 amyloid induced cell death^54^.

### Binding mode of vitamin k3 to Aβ-42 peptide

Molecular docking study was performed to get insight into the type of interactions involved between vitamin k3 and Aβ-42 that are responsible for aggregation inhibition. Docking results are summarized in supplementary Figure S5 and supplementary Table 3. Figure S5b shows that vitamin k3 interacts with six amino acids residues of Aβ-42 including Ser^8^, Glu^11^, Val^12,^ Gln^15^, Lys^16^ and Phe^19^. Vitamin k3 interact with Aβ-42 with Ser^8^, Glu^11^, Val^12,^ Gln^15^ and Phe^19^ via hydrophobic interactions. Further hydrogen bonding is involved between Lys^16^ of Aβ-42 and vitamin k3. From these results it can be inferred that Val^12^, Gln^15^, Lys^16^ are common residues that are generally involved in the interaction of other inhibitors like Myricetin, EGCG, Curcumin and Pyrazinamide which suggests that vitamin k3 may follow the same mechanism of action against amyloidogenesis^55^,^56^,^57^. Recently, Sinha *et al*. reported that the molecular tweezer (CLR01) a Lys-specific synthetic compound prevented cytotoxicity and oligomerization of Aβ-42 through non-covalent interaction and their study advocates the crucial role of Lys16 and Lys28 in Aβ-42 aggregation^58^.

## Conclusion

To conclude, current study reports that vitamin k3 is a potent therapeutic molecule that inhibits the progression of HEWL and Aβ-42 aggregation process by exploration of various biophysical and imaging tools including ThT fluorescence, ANS binding, CD measurements, DLS and TEM. Vitamin k3 inhibits the aggregation process starting from very beginning of nucleation phase. The non fibrillar aggregates formed in the presence of vitamin k3 contain less β-sheet structures as obtained from CD results. Sparsely populated and smaller sized aggregates were formed in presence of vitamin k3 as revealed by TEM and DLS results. Further, vitamin k3 which seems to be therapeutic via conventional biophysical and imaging techniques also imparts beneficial effects in reducing cytotoxicity in human neuronal cell line. Hydrophobic interactions are key forces involved between HEWL and vitamin k3 whereas in case of Aβ-42 vitamin k3 complex both hydrogen bonding as well as hydrophobic interactions are involved, implicated to play a dominating role in the inhibiting activity. The future projections of this study are to investigate the modulatory role of not only vitamin k3 but also its related molecules in disease progression in systemic amyloidosis and Alzheimer disease mice models.

## Methods

### Materials

Hen egg white lysozyme (HEWL), Aβ-42 peptide, Thioflavin T (ThT), 1-anilino 8 naphthalene sulphate (ANS) and vitamin k3 were procured from Sigma Aldrich, India. All other reagents used were of analytical grade.

### pH measurements

pH measurements were carried out using Mettler Toledo Seven Easy pH meter (model S20) which was routinely calibrated with standard buffers. The experiments were performed at the 20 mM pH 7.4 sodium phosphate buffer. All preparations used in the experiments were filtered through 0.45 μm Millipore Millex-HV PVDF filter.

### Sample preparation

A stock solution of HEWL was made in 20 mM phosphate buffer pH 7.4 and extensively dialyzed against the same buffer and concentration was determined using a UV-visible spectrophotometer (Perkin Elmer Lambda 25) 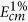
= 12.4 at 280 nm. For preparation of amyloid, 100 μM of HEWL was used and samples were incubated at 65 °C in the presence of 50 mM NaCl for 120 hours in a circulating shaking water bath. Aliquots were taken from the stock at different time intervals. The Aβ-42 was prepared as described in literature^59^. For preparation amyloid 100 μM of Aβ-42 was kept at 37 °C and incubated for 70 hours under constant stirring in absence and presence of 50 and 100 μM of vitamin k3. For further analysis, aliquots were taken from each set at different time intervals.

### ThT fluorescence spectroscopy measurements

ThT fluorescence assay was performed with Shimadzu fluorescence spectrophotometer (RF-5301 PC). ThT stock solution was prepared in double distilled water and filtered with 0.2 micron millipore filter. HEWL samples incubated with or without vitamin k3, from each set samples were withdrawn at definite interval of time and mixed with ThT to accomplish final protein and dye concentration of 15 μM. Aβ-42 samples incubated with or without vitamin k3, from each set samples were withdrawn at definite interval of time and mixed with ThT to accomplish final protein and dye concentration of 30 μM. Samples were incubated in dark for 30 minutes. The ThT was excited at 440 nm and spectra were recorded from 450 to 600 nm. The excitation and emission slit widths were set at 5 and 10 nm, respectively. Sodium phosphate buffer 20 mM (pH 7.4) was used for dilution and spectra were corrected from respective blanks. All measurements were performed in triplicates. All curves were fitted as we described previously^43^.

### Congo red binding assay

Congo red was dissolved in a 20 mM phosphate buffer (pH 7.4) consisting of 50 mM NaCl and filtered through 0.45 μM membrane filter. The concentration was determined using ε_M_ 45,000 M^−1^ cm^−1^ at 498 nm. The HEWL and Aβ-42 concentration were fixed at 15 and 30 μM respectively. CR and protein were mixed at a molar ratio of 1:1 in the absence and presence of vitamin k3 and kept for 30 minutes. The absorbance spectra (400–650 nm) of the samples were recorded with a UV-Visible spectrophotometer (Perkin Elmer Lambda 25) in a 1 cm path length cuvette.

### ANS fluorescence measurements

HEWL samples (15 μM) in absence and presence of vitamin k3 were mixed with 50 fold molar excess of ANS and then mixtures were kept in dark for 30 minutes at room temperature. Aβ-42 (30 μM) sample in absence and presence of vitamin k3 was mixed with 50 fold molar excess of ANS and then mixtures were kept in dark for 30 minutes at room temperature. ANS fluorescence intensities were recorded with excitation at 380 nm and emission between 400 to 600 nm on a fluorescence spectrophotometer (RF-5301 PC). Excitation and emission slit width were set at 5 nm. All measurements were performed in triplicates.

### Far-UV circular dichroism measurements

The circular dichroic measurements were performed on a JASCO spectropolarimeter (J-815) with a thermostatically controlled cell holder attached to a peltier with multitech water circulator. The experiments were carried out with HEWL (15 μM) and Aβ-42 (30 μM) in absence and presence of vitamin k3. Spectra were scanned in the range of 200–250 nm in a cuvette of 0.1 cm path length with scanning speed of 100 nm/min. Each spectrum was an average of three scans.

### Dynamic light scattering (DLS) measurements

The change in aggregation behaviour of HEWL and Aβ-42 in the presence of varying concentration of vitamin k3 were determined using DLS. The hydrodynamic radii (*R*_*h*_), measurements were done using a protein concentration of 10 μM at 830 nm on a DynaPro-TC-04 dynamic light scattering instrument (Protein Solutions, Wyatt Technology, Santa Barbara, CA) equipped with a temperature controlled microsampler. All solutions were filtered through a 0.22 μM pore sized micro filter (Whatman International, Maidstone, UK). The measured hydrodynamic radius (*R*_*h*_) was the average of 50 measurements. The mean *R*_*h*_ and polydispersity (Pd) were estimated, on the basis of an autocorrelation analysis of scattered light intensity based on the translational diffusion coefficient, from the Stokes–Einstein equation^60^


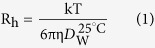


where *R*_*h*_ is the hydrodynamic radius (nm), k is the Boltzmann’s constant, T is the absolute temperature (K), η is the viscosity of water and 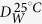 is the translational diffusion coefficient. All the samples were incubated for 120 hour at 65 °C prior to measurements.

### Transmission electron microscopy (TEM)

TEM images were taken on Philips CM-10 transmission electron microscope operating at an accelerating voltage of 200 kV. The amyloid fibril formation was assessed by applying 6 μL of HEWL (15 μM) and Aβ-42 (30 μM) in absence and presence of vitamin k3 on 200-mesh copper grid covered by carbon-stabilized formvar film. Excess of fluid was removed after 2 min and the grids were then negatively stained with 2% (w/v) uranyl acetate. Images were viewed at 10,000X.

### Cell culture

SH-SY5Y (human neuroblastoma cell line) cells were cultured in DMEM medium in humidified 5% (v/v) CO_2_/air at 37 °C in 10% (v/v) (fetal bovine serum) FBS and 100 U/ml penicillin.

### Cell viability assay

MTT (3, (4, 5-dimethylthiazol-2-yl) 2, 5-diphenyltetrazolium bromide) reduction assay was used to measure the cell viabilities of SH-SY5Y. MTT, in the presence of viable cells reduce to form blue formazon crystals, toxicity leads to inhibition of formazon production^26^. For the MTT reduction assays, sample solutions of HEWL and Aβ-42 in absence and presence of vitamin k3 were added to the SH-SY5Y cells in the 96-well plates. Cells were seeded at 5,000 cells/well on 96-well plates and incubated for 24 hours before the treatment. The HEWL and Aβ-42 sample solutions (incubated over a period of 120 hours and 70 hours at 65 °C and 37 °C respectively) were incubated with SH-SY5Y cells for 24 hours, and MTT reduction was performed. MTT was added to the culture medium to yield a final concentration of 0.5 mg/ml and incubated for 4 hours at 37 °C in CO_2_ incubator then removed supernatant carefully, 200 μL of DMSO was added and mixed. After 20 hours of incubation in a humidified CO_2_ incubator, the absorbance at 585 nm was read using a Micro plate absorbance reader (Bio-Rad Instruments, iMark^TM^). Cell viability was compared to control cells without prior exposure to the fibril solutions.

### Molecular docking study of HEWL-vitamin k3 interaction

The molecular docking study was performed using Autodock 4.2 and Autodock tools (ADT) using Lamarckian genetic algorithm. The crystal structure of HEWL (PDB id: 2LYZ) and Aβ-42 (PDB id: 1IYT) were obtained from Brookhaven Protein Data Bank. Three dimensional structure of vitamin k3 (CID: 4055) was obtained from PubChem. Water molecules, ions were removed and all hydrogen atoms were added. Then partial Kollman charges were assigned to protein. The protein was set to be rigid and there was no consideration of solvent molecules on docking. The grid size was set to be 116, 116 and 116 along X, Y and Z axes with 0.522 Å grid spacing for HEWL- vitamin k3. But in case of Aβ-42- vitamin k3 grid size was set to be 60, 60 and 60 along X, Y and Z axes with 0.426 Å grid spacing. Auto dock parameters used were GA population size: 150 and maximum number of energy evolutions: 250,0000. 10 best solution based on docking score was retained for further analysis, Discovery studio 3.5 were used for visualization and for the identification of residues involved in binding.

### Statistical analysis

All data were presented as mean ± standard deviation from 3 independent determinations. The statistical analysis was made by performing one-way ANOVA for 3 independent determinations. Significance of results was determined as p ≤ 0.01, unless otherwise stated.

## Additional Information

**How to cite this article**: Alam, P. *et al*. Vitamin k3 inhibits protein aggregation: Implication in the treatment of amyloid diseases. *Sci. Rep.* **6**, 26759; doi: 10.1038/srep26759 (2016).

## Supplementary Material

Supplementary Information
