# Supplementary material for: Vitamin k3 inhibits protein aggregation: Implication in the treatment of amyloid diseases
Source: Sci Rep. 2016 May 27;6:26759. doi: 10.1038/srep26759 (PMC4882616; doi:10.1038/srep26759)
Supplement: Supplementary Information [file srep26759-s1.pdf]

Research Article

Vitamin k3 inhibits protein aggregation: Implication in the treatment of amyloid diseases

Parvez Alam[1], Sumit Kumar Chaturvedi[1], Mohammad Khursheed Siddiqi[1], Ravi Kant Rajpoot[2], Mohd Rehan Ajmal[1], Masihuz Zaman[1] and Rizwan Hasan Khan[1]*

[1]Molecular Biophysics and Biophysical Chemistry Group, Interdisciplinary Biotechnology Unit, Aligarh Muslim University, Aligarh-202002, India

[2]Recombinant Gene Product Group, International Centre for Genetic Engineering and Biotechnology, Aruna Asaf Ali Marg, New Delhi- 110067, India

.

*To whom correspondence should be addressed

Prof. Rizwan Hasan Khan
Interdisciplinary Biotechnology Unit,
Aligarh Muslim University,
Aligarh-202002, U.P., India.

E-mail:rizwanhkhan@hotmail.com,
rizwanhkhan1@gmail.com

Phone: 91-571-2720388

Fax: + 91-571-2721776

Supplementary information

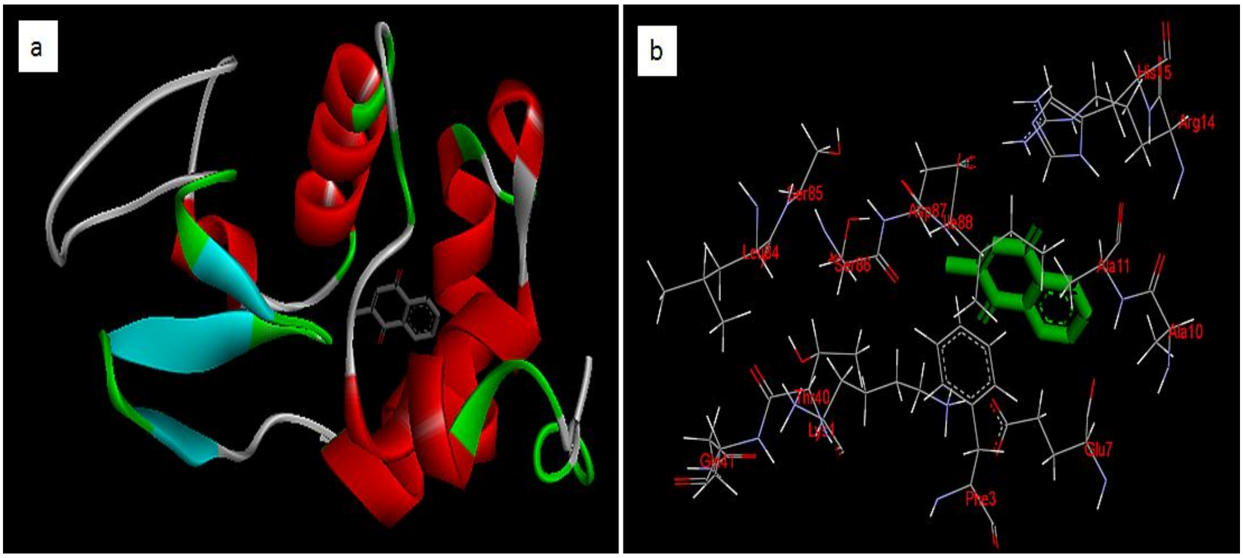

Supplementary figure S1: Molecular Docking results of HEWL-vitamin k3 complex. (a) Vitamin k3 is shown in a stick representation, and HEWL represented with ribbon model. (b) Detailed view of the docking poses of HEWL- vitamin k3 complex.

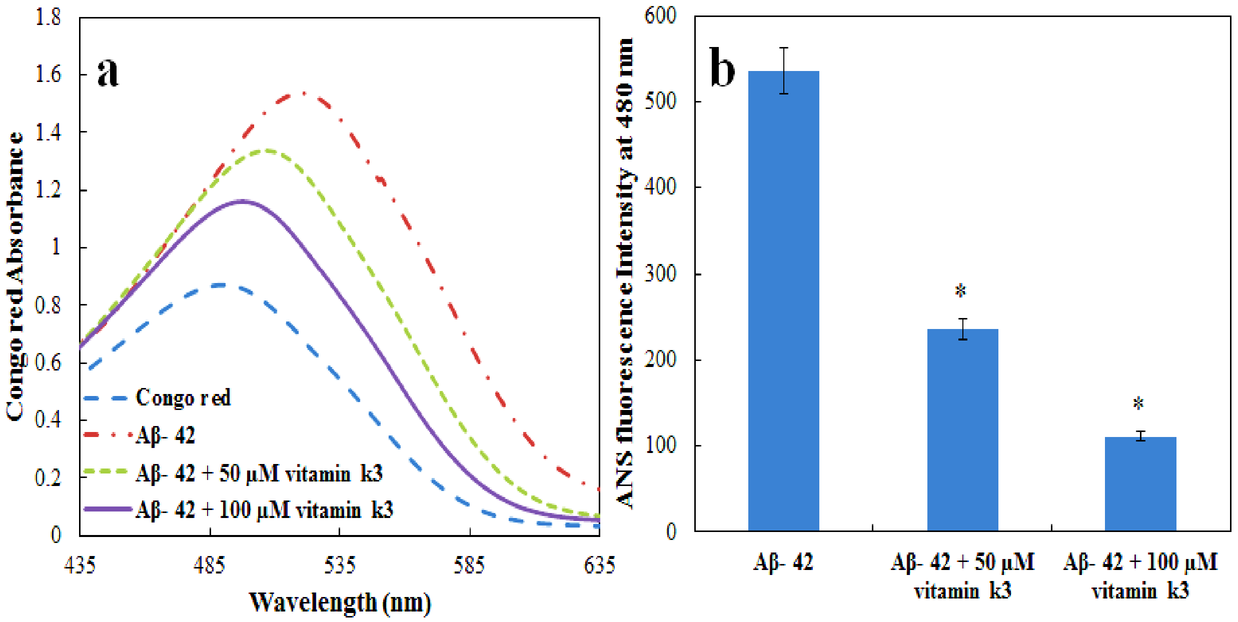

Supplementary figure S2: (a) Congo red binding absorption spectra of Aβ- 42 in the absence and presence of vitamin k3. Samples were incubated with two different concentration of vitamin k3 (50 and 100 µM) at 37 ºC for 70 hours. (b) ANS fluorescence intensity of Aβ- 42 at 480 nm incubated at 37 ºC for 70 hours in absence and presence of vitamin k3 (50 and 100 µM). *Statistically different from the Aβ- 42 $p \leq 0.01$.

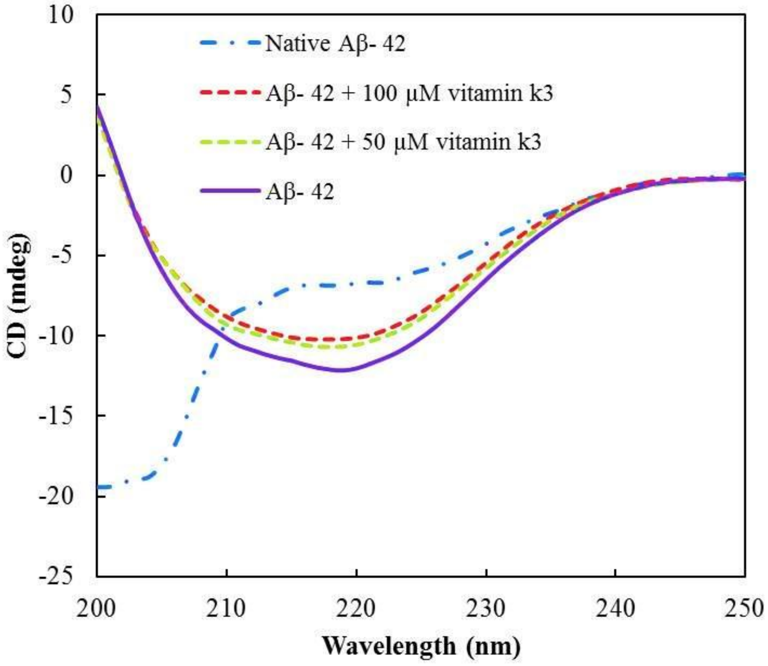

Supplementary figure S3: Far-UV CD spectra of Aβ- 42 in absence and presence of vitamin k3. Native Aβ-42 without incubation and Aβ-42 incubated for 70 hours at 37 °C.

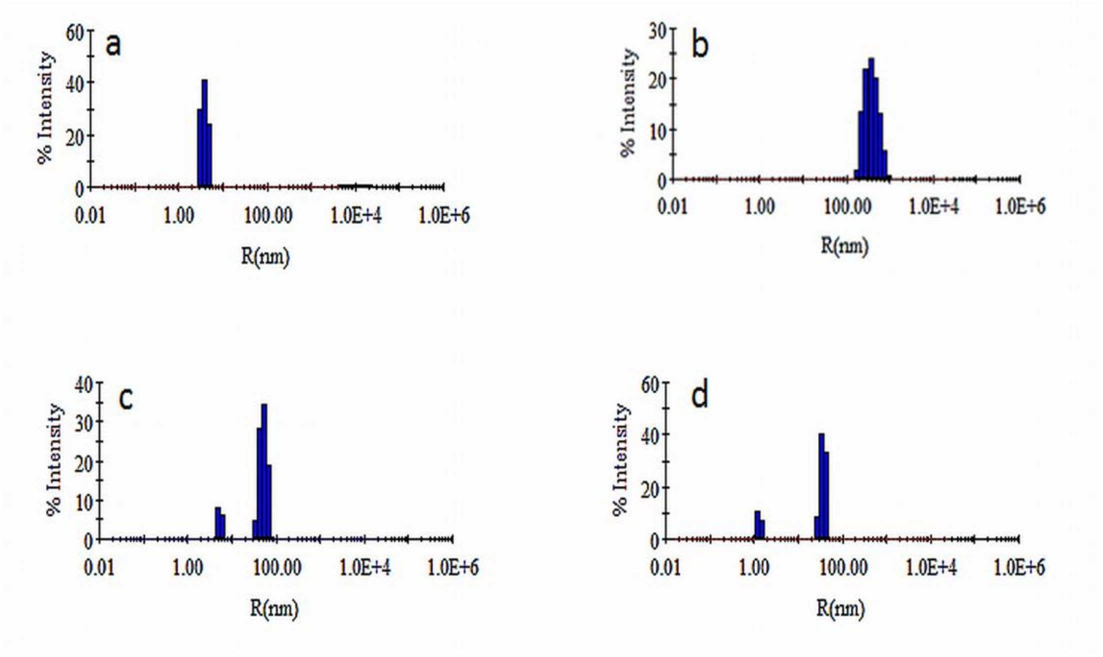

Supplementary figure S4: DLS pattern of Aβ- 42 in absence and presence of vitamin k3 (a) Native Aβ- 42 and after incubation at 37 ºC over a period of 70 hours (b) Aβ- 42 (c) Aβ- 42 + 50 μM vitamin k3 and (d) Aβ- 42 + 100 μM vitamin k3.

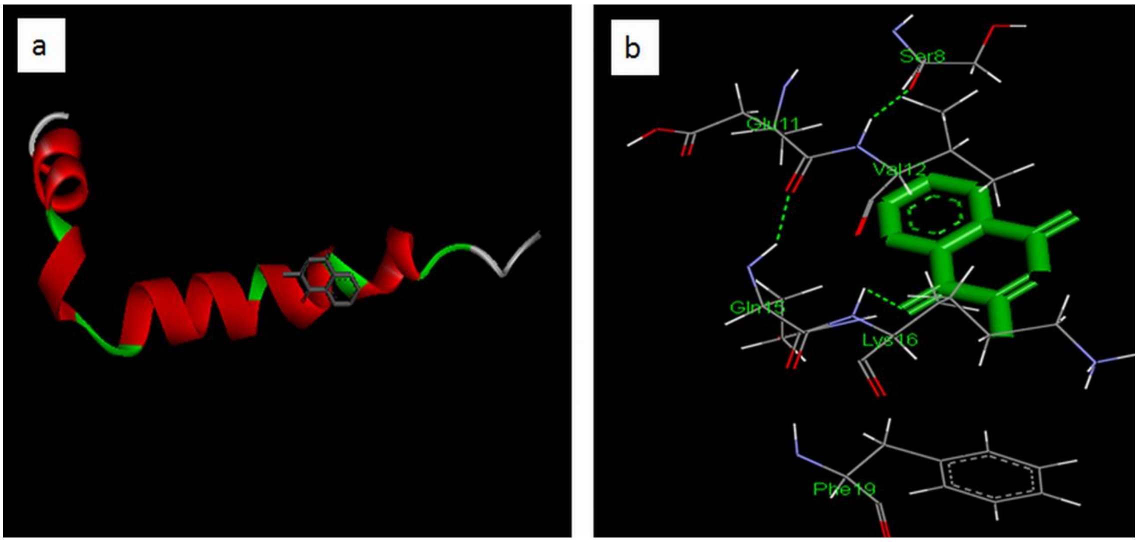

Supplementary figure S5: Molecular docking results of Aβ-42+vitamin k3 complex. (a) Vitamin k3 is shown in a stick representation, and Aβ-42 represented with ribbon model. (b) Detailed view of the docking poses of Aβ-42+vitamin k3 complex.

Supplementary Table 1: Changes in secondary structural content of HEWL in absence and presence of vitamin k3

	HEWL			HEWL+50 µM vitamin k3			HEWL+100 µM vitamin k3		
Time (hours)	α [%]	β [%]	Other [%]	α [%]	β [%]	Other [%]	α [%]	β [%]	Other [%]
0	32	12	56	37	12	51	41	11	48
120	21	37	42	29	25	46	31	19	50

Supplementary Table 2: Molecular docking parameters of HEWL-vitamin k3 interaction.

**Amino acid residues**	**Interaction involved**	**Binding energy (kcal M^{-1})**
Lys^{1}	Hydrophobic	-3.98
Phe^{3}		
Glu^{7}		
Ala^{10}		
Ala^{11}		
Arg^{14}		
His^{15}		
Thr^{40}		
Gln^{41}		
Leu^{84}		
Ser^{85}		
Ser^{86}		
Asp^{87}		
Ile^{88}		

Supplementary Table 3: Molecular docking parameters of Aβ- 42 and vitamin k3 interaction.

Amino acid residues	Interactions involved	Binding energy (kcal M^{-1})
Ser[8]	Hydrophobic	-4.15
Glu[11]	Hydrophobic	
Val[12]	Hydrophobic	
Gln[15]	H- Bonding	
Lys[16]	Hydrophobic	
Phe[19]	Hydrophobic	
